# Supplementary material for: Critical mechanistic features of HIV-1 viral capsid assembly
Source: Sci Adv. 2023 Jan 6;9(1):eadd7434. doi: 10.1126/sciadv.add7434 (PMC9821859; doi:10.1126/sciadv.add7434)
Supplement: Supplementary file 1 — Supplementary Text Figs. S1 to S4 [file sciadv.add7434_sm.pdf]

Science Advances
AAAS

Supplementary Materials for

Critical mechanistic features of HIV-1 viral capsid assembly

Manish Gupta *et al.*

Corresponding author: Gregory A. Voth, gavoth@uchicago.edu

Sci. Adv. **9**, eadd7434 (2023)
DOI: 10.1126/sciadv.add7434

The PDF file includes:

Supplementary Text
Figs. S1 to S4

Other Supplementary Material for this manuscript includes the following:

Movies S1 and S2

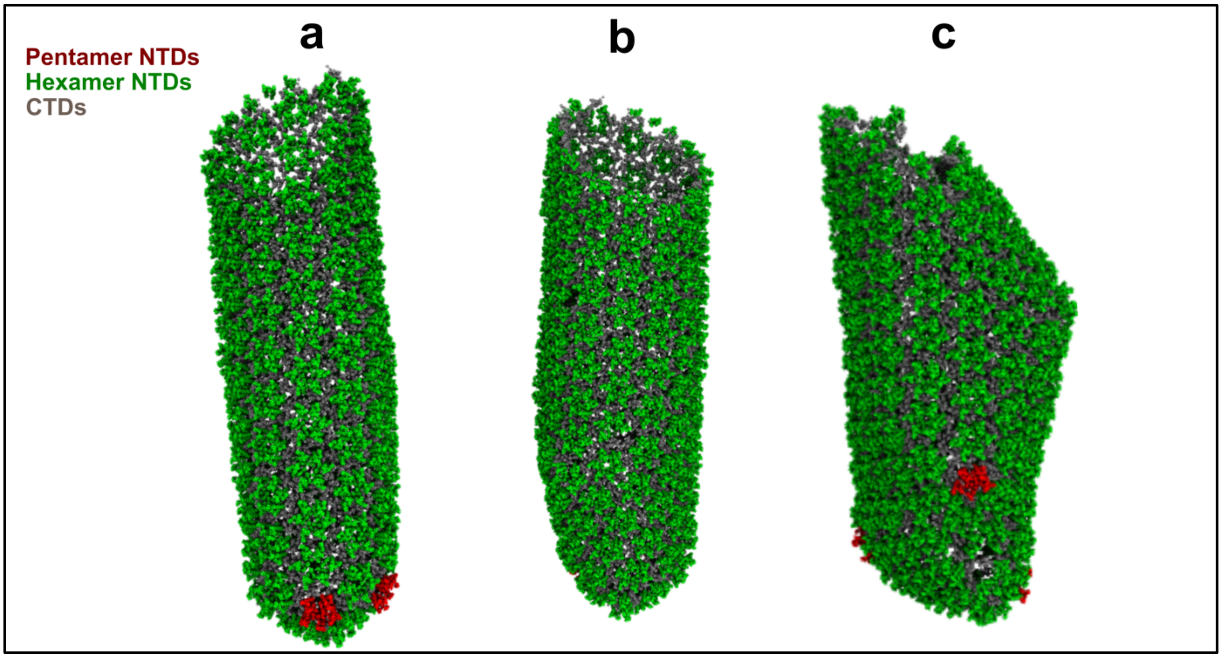

Fig. S1. CGMD simulations of CA dimer assembly in the absence of IP6. Snapshots of final structures from three additional CGMD simulations of CA assembly in the absence of IP6 (CA

hexamer NTDs are green, pentamer NTDs are red and all CTDs are gray). Stable pentamers are embedded in the lattice. In panel (c) a partially conical capsid is formed.

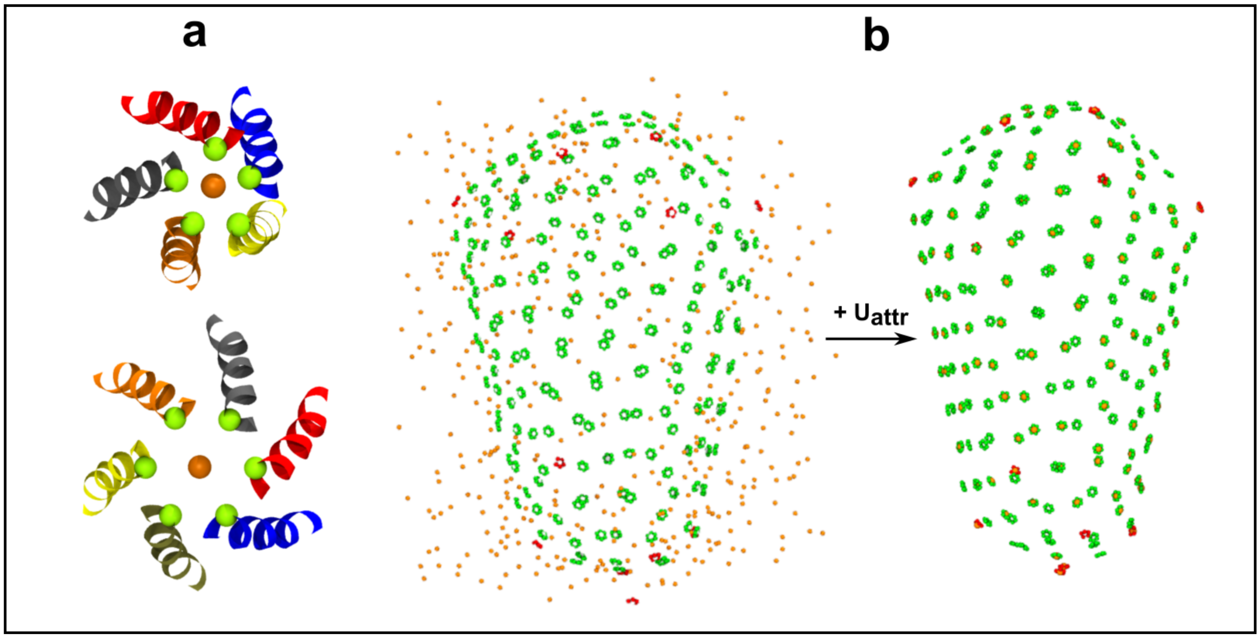

Fig. S2. Schematic of IP6 binding with pre-assembled capsid, during IP6 parameterization. (a) CG representation of the R18 ring (green beads) and IP6 (orange bead) in hexamers and pentamers. Helix 1 of the CA hexamer (from PDB 3H4E (*57*)) and pentamer (from PDB 3P04 (*15*)) shown in distinct colors to highlight the binding location of IP6 in the CG model. (b) IP6 binding to R18 rings throughout the pre-assembled capsid. Only R18 bead (green and red for the hexamer and pentamer, respectively) bound IP6 (orange beads) are shown for clarity.

Supplementary Text

Intrinsic curvature of the capsomers

It has been shown that capsomers in HIV-1 capsids are intrinsically curved (*40*). We analyzed our tubular and conical capsids to understand the intrinsic flexibility of these structures. To determine the planar deviation of each CA in the capsomers, we considered a vector from the center of mass (COM) of CTD to NTD in each CA and determined the relative angle it forms with the average normal vector of that capsomer. Now, in a perfectly flat symmetric hexamer or pentamer, this angle should be zero. But, we observed that both pentameric (Fig. S3a) and hexameric (Fig. S3b) capsomers in the conical capsid deviated from ideal planar structures reflecting intrinsic curvature. We note that pentamers in HIV-1 cones adopt to a wide distribution of curvatures whereas hexamers adopt a homogenous distribution of curvatures (Fig. S3b) deviating from planarity. We also analyzed hexamers from CA tubes (Fig. S3c) which also have a homogenous distribution of intrinsic curvature.

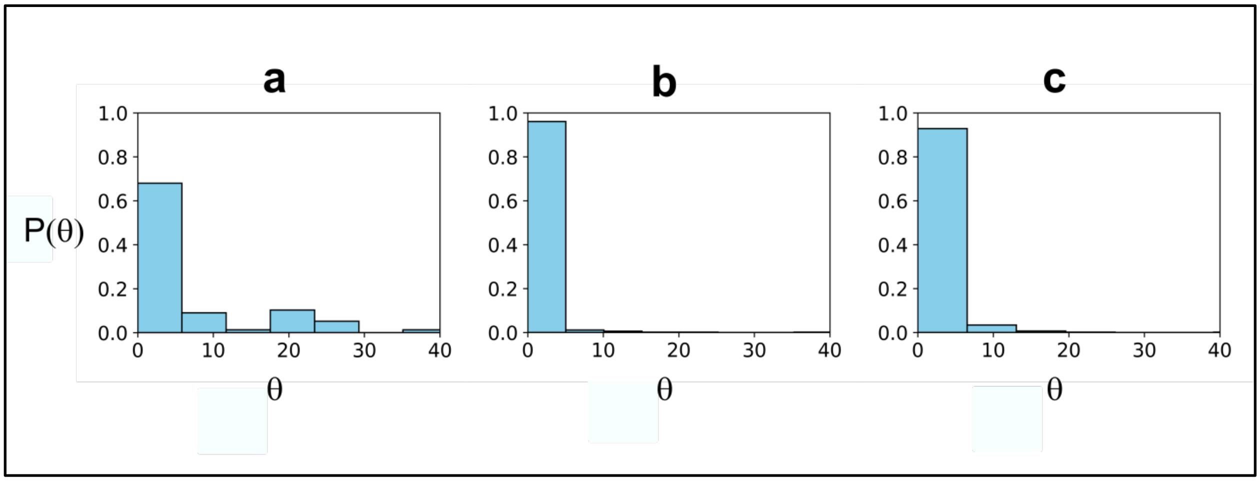

Fig. S3. Curvature distribution of CA in capsomers. (a) Curvature distribution of pentamers in CA conical capsid. (b) Curvature distribution of hexamers in CA conical capsid. (c) Curvature distribution of hexamers in CA tube.

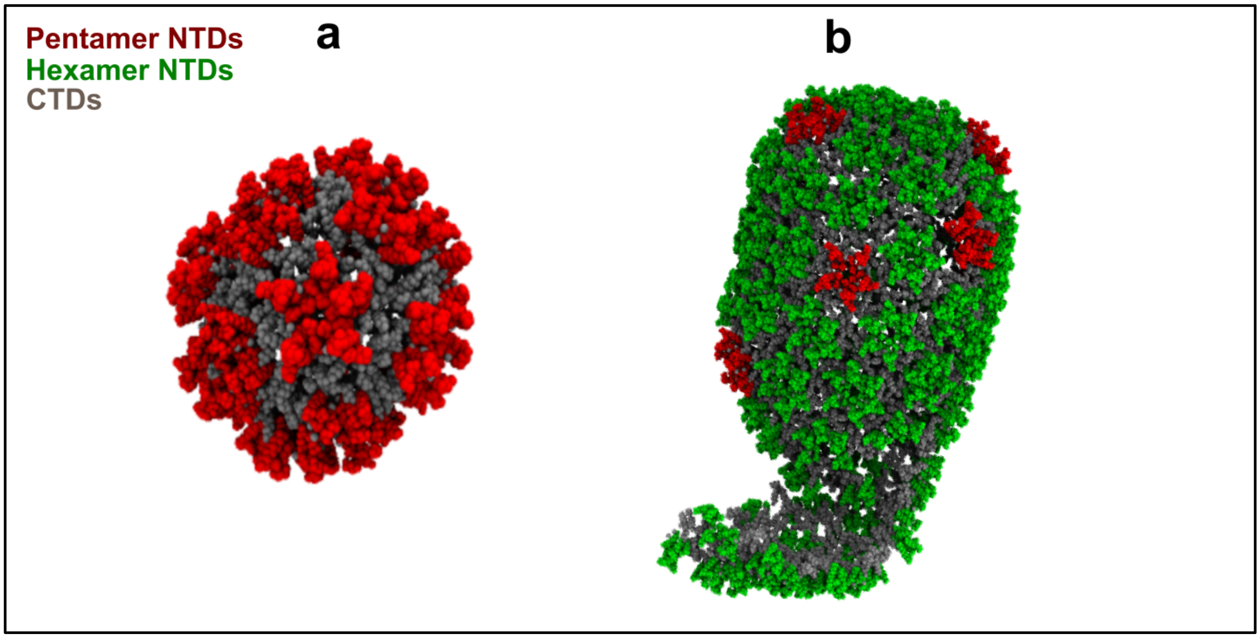

Fig. S4. Non-canonical capsid like particles (CLP). (a) T=1 icosahedral particle formed exclusively when IP6-R18 interaction strength was increased. (b) Lamellar CLP (*46*) was observed for 5:1 IP6/CA simulation (CA hexamer NTDs are green, pentamer NTDs are red and all CTDs are gray).
